# The importance of communication in promoting voluntary participation in an experimental trial: A qualitative study based on the assessment of the gamma-interferon test for the diagnosis of bovine tuberculosis in France

**DOI:** 10.1371/journal.pone.0185799

**Published:** 2017-10-03

**Authors:** Clémence Boireau, Barbara Dufour, Anne Praud

**Affiliations:** 1 École Nationale des Services Vétérinaires, VetagroSup, Marcy L’Etoile, France; 2 Université de Lyon, French Agency for Food, Environmental and Occupational Health & Safety (ANSES), Laboratoire de Lyon, Unité Épidémiologie, Lyon, France; 3 École Nationale Vétérinaire d’Alfort, Epidemiology of Animal Infectious Diseases Unit, French Agency for Food, Environmental and Occupational Health & Safety (ANSES), Paris-Est University, Maisons-Alfort, France; Atlantic Veterinary College, CANADA

## Abstract

Understanding the factors leading each stakeholder to participate in an experimental trial is a key element for improving trial set-up and for identifying selection bias in statistical analyses. An experimental protocol, validated by the European Commission, was developed in France to assess the ability of the gamma-interferon test in terms of accuracy to replace the second intradermal skin test in cases of suspected bovine tuberculosis. Implemented between 2013 and 2015, this experimental trial was based on voluntary participation. To determine and understand the motivation or reluctance of farmers to take part in this trial, we carried out a sociological survey in France. Our study was based on semi-structured interviews with the farmers and other stakeholders involved. The analysis of findings demonstrated that shortening the lock-up period during tuberculosis suspicion, following the use of a gamma-interferon test, was an important aim and a genuine challenge for the animal health stakeholders. However, some farmers did not wish to continue the trial because it could potentially have drastic consequences for them. Moreover, misunderstandings and confusion concerning the objectives and consequences of the trial led stakeholders to reject it forcefully. Based on our results, we offer some recommendations: clear and appropriate communication tools should be prepared to explain the protocol and its aims. In addition, these types of animal health trials should be designed with the stakeholders’ interests in mind. This study provides a better understanding of farmer motivations and stakeholder influences on trial participation and outcomes. The findings can be used to help design trials so that they promote participation by farmers and by all animal health stakeholders in general.

## Introduction

### Origins and features of the French experimental protocol to assess the gamma-interferon test

Bovine tuberculosis (bTB) is an infectious and chronic disease caused by *Mycobacterium bovis* [1, 2]. It is generally asymptomatic and difficult to detect in live animals. In the European Union, only cervical skin tests (STs), including the single intradermal tuberculin test and the comparative cervical tuberculin test, are currently recommended for the diagnosis of bTB in live cattle. The testing programme for bTB in France is conducted annually in compliance with European Directive 64/432/EEC, and covers all cattle older than 24 months of age. Field testing is performed by private-practice veterinarians. Two types of STs are used: the single intradermal tuberculin test (SIT) and the single intradermal comparative cervical tuberculin test (SICCT). The test has one of three outcomes: negative, doubtful or positive, and the results can be interpreted at different levels of severity depending on the ST used (SIT or SICCT). Nevertheless, STs are neither fully sensitive nor specific [3]. For this reason, after an initial non-negative (i.e. positive or doubtful) result by SIT screening or after an initial doubtful result by SICCT screening (suspected case), a second skin test (SST) is performed to confirm or rule out bTB suspicion. However, the SST can only be carried out 42 days after the initial ST, due to a desensitisation phenomenon of six weeks (S1 Fig). During this period, suspect herds have to be locked up: products cannot be sold and all movements of cattle are forbidden. Cattle that retest positive are removed from the farm and slaughtered to confirm the infection, without financial compensation. It is important to note that infection can only be confirmed *post-mortem*, after slaughtering some suspect animals and performing PCR and sample cultures. If the infection is confirmed, the entire herd is slaughtered and the farmer receives financial compensation to reconstitute the herd. If the culture or PCR rule out infection, cattle that remain are retested every six weeks until the farm is believed to be free of bTB.

The gamma-interferon (IFN) test is an alternative test to detect bTB [3]. The advantage is that it can be used in series in the days just after non-negative ST results, avoiding the six-week lock-up period. The use of IFN for bTB has not been widely studied in the literature [4, 5] and is not authorised by European Directive 64/432/EEC. To assess the accuracy of the IFN test used immediately after a non-negative result, an experimental protocol (EP), validated by the European Commission, was developed in France for implementation between 2013 and 2015. The general aim of this protocol was to assess whether the SST performed six weeks after a first non-negative ST result can be replaced by an IFN test performed just days after the first skin test [6].

**Fig 1:**
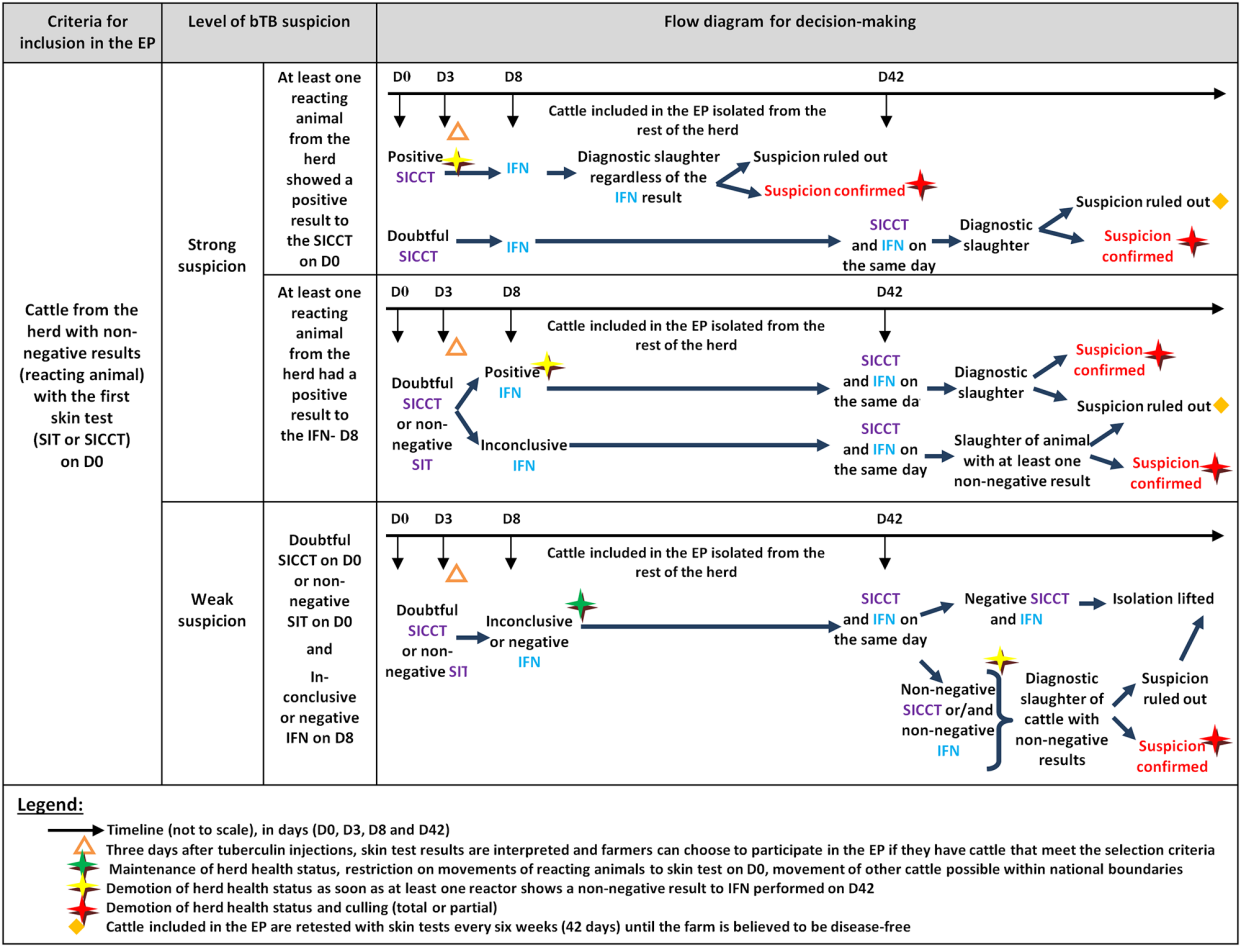
Diagrams illustrating the management procedures in the experimental protocol.

The inclusion of farms in the EP took place on a voluntary basis: as soon as farmers had cattle with non-negative ST results they were eligible to participate in the study [7]. The farmers were offered no financial incentive to participate. Fig 1 provides an overview of the experimental protocol. Contrary to the standard protocol, the EP allowed cattle movements and sale of cattle products in France, provided that the first non-negative ST was an SIT, or an SICCT with a doubtful result, and that the IFN test performed just days after was negative or inconclusive for all reacting animals (weak suspicion in Fig 1). On the other hand, in accordance with the EP, the decision to slaughter was based not only on the results of the ST, but also on the IFN test results. This potentially increased the probability of enforced slaughter.

### General purpose of the sociological study and study goals

Here, we set out to explore the factors influencing farmer participation in the experimental protocol. Our study aimed to discover the views of the farmers on IFN, its use, and how their opinion could affect their decision to participate or not. The EP constituted an alternative management option for suspect bTB cases. However, choosing an alternative method can affect the relationships and interactions between stakeholders [8, 9]. Therefore, introducing a change in the standard procedure raises the question of the importance of the factors that influence decision-making with regard to joining the EP, and those that affect the interactions between stakeholders. Ultimately, our study aimed to provide a better understanding of farmer motivations and stakeholder influences around this type of trial, to help researchers or animal health managers design trials that promote farmer and other stakeholder involvement.

## Methods

### Study design

#### Location and time

The choice of the study area was based on three criteria. First, the area should correspond to a French administrative division (department) because the bTB management procedure is coordinated at this level by the departmental veterinary services. Second, the department must implement bTB screening, but the IFN test must never have been used in the past for bTB screening, because previous use of this test may influence the opinions of potential participants. Third, the department must allow farmers to participate voluntarily in the EP (in a few departments, participation was enforced by a prefectural order) [10]. Based on these criteria, the French department Ardennes, located in north-eastern France was selected. The study period coincided with the two bTB screening campaigns of 2013–2014 and 2014–2015, during which the EP was proposed and performed.

#### Target population and pre-selection of participants

**Fig 2:**
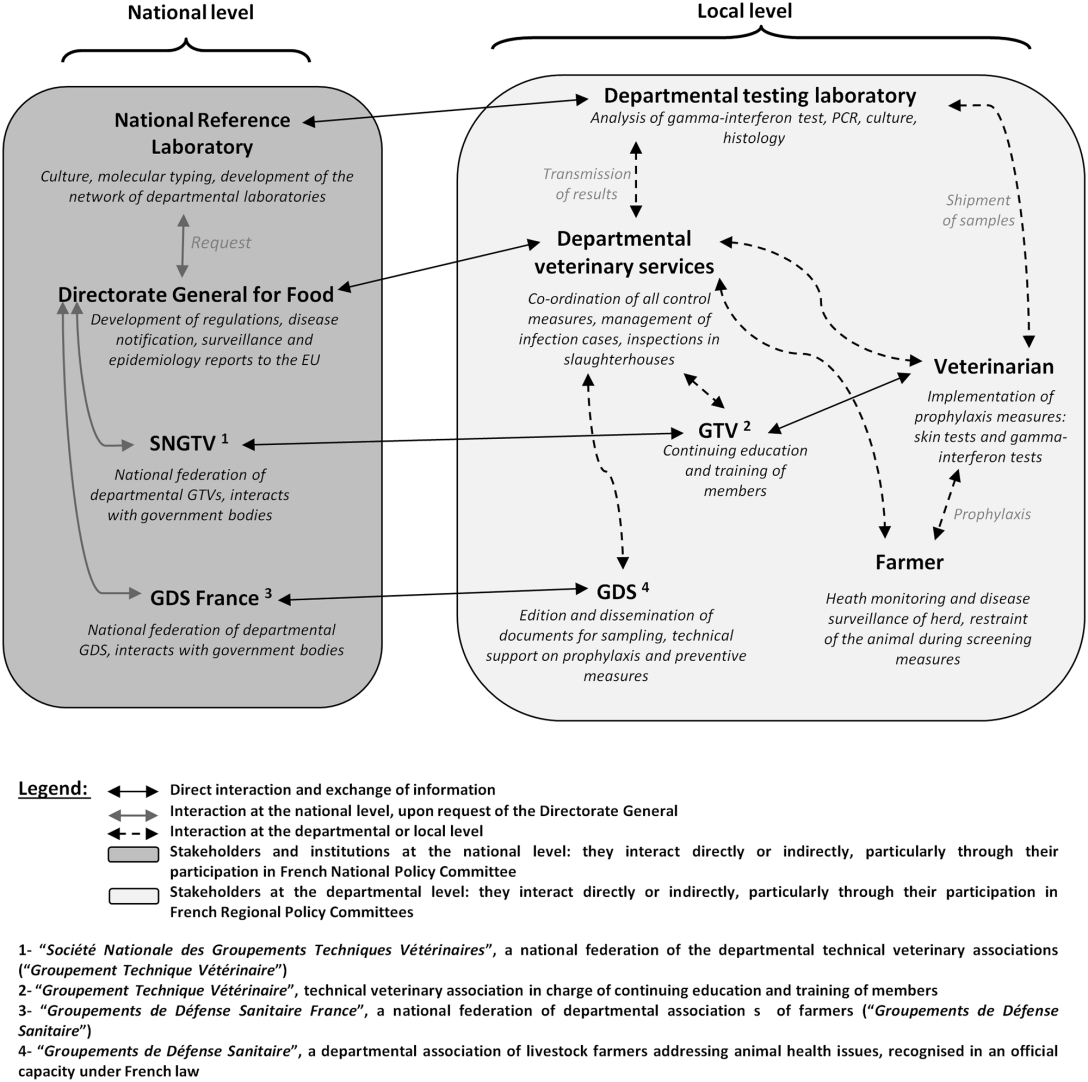
Schematic representation of the network of stakeholders involved in the management of bovine tuberculosis.

The target population included all stakeholders involved in the EP at the local scale: farmers, veterinarians, representatives of the animal health protection federation GDS (*Groupements de Défense Sanitaire)*, a departmental association of livestock farmers addressing health issues, recognised in an official capacity under French law, representatives of the technical veterinary association GTV (*Groupement Technique Vétérinaire)*, the administrative competent authority represented by veterinary services (inspectors and technicians), and the departmental testing laboratory. The French veterinary services, farmers, official veterinarians, GTV, GDS and the testing laboratory all already interact in various, pre-defined ways (see network illustrated in Fig 2).

Three main criteria were used to collect the points of views of all stakeholders: their representativeness (linked to their eligibility), their availability, and randomness. Firstly, stakeholder representativeness was met if they belonged to one of the six types of stakeholders [11]. More specifically, farmers were pre-selected taking into account their participation in the EP (according to the eligibility criteria described above). In Ardennes, 12 farmers took part in the EP during the first campaign (2013–2014) among 15 farmers who met the eligibility criteria. During the second campaign (2014–2015), 4 farmers joined the EP among 14 who met the eligibility criteria. Some farmers were eligible for both campaigns, but none participated in both. Thus in total, 16 different farmers participated in the EP. They were listed and then randomly selected to be interviewed.

For the five other types of stakeholders, eligibility criteria took into account the field of expertise and involvement in the EP. Veterinarians were eligible if the farmer they followed also participated in the EP. Veterinary agents involved in EP management, laboratory technicians in charge of IFN analyses, and representatives of the GTV or GDS involved in the EP were selected. Therefore, selection was based on expertise and competence, looking for “*key informants*” [12, 13]. Participation in the EP significantly narrows the subject and relevant information could not be collected from randomly selected informants [11].

The present study did not require formal consent and approval by the *Comité de protection des personnes* (French ethics committee) because it was not a clinical trial. The selected participants were contacted individually by telephone (using a call guide) to provide information on the purpose, nature and background of the study. Specifically, potential participants were advised that the study involved seeking their views and thoughts on the EP and why they participated or not. Participants were informed that their opinions and comments would remain anonymous, and that any material potentially leading to individual identification would be removed. It was made clear that by agreeing to be interviewed, participants agreed to be part of the study. Once verbal consent was obtained [14], a time and place were arranged for an individual interview to be conducted. All stakeholders contacted eventually agreed to be interviewed, even though some farmers were reluctant at first.

### Data collection

An interview is an effective method of obtaining information through open-ended discussions. It is appropriate for studying attitude, conception, belief, experience, knowledge, values, and standards, which are difficult to observe directly [14–17]. Therefore, an interview is a structured and tactical conversation that supports a communication aim [16, 17]. Leaving the interviewee free to formulate answers to thematic questions, the semi-structured interview allows for more flexibility than a questionnaire [18]. A semi-structured interview also establishes a determined and fixed framework created by the interviewer [19, 20].

To maximise both the quantity and quality of data collected, interview guides were elaborated by the authors for each type of stakeholder [21]. These guides (Table 1 and S1 Table) were submitted to experts for an opinion and then pre-tested in an exploratory interview. This strategy produced effective interview guides [17] used as soon as the survey started. Nevertheless, after the first interviews, the guides were revised slightly and the order in which the questions were asked varied between interviews. In addition, the interviewer asked follow-up questions to uncover further details and to delve into the participants’ individual responses [18]; these questions evolved with subsequent interviews.

**Table 1:** Topics and underlying topics in the guide for interviews with farmers.

Topic	Underlying topic
**Opening question**	What led you to join the experimental protocol?
**Knowledge regarding the experimental protocol**	Ability to describe the experimental protocol
	Knowledge regarding the purpose of the trial
**Managing temporary change**	Presentation of the experimental protocol to farmers (who, when, where)
	Information received: relevance, quantity, quality
	Information relayed in the field
	Active research of further information (what, why, where, when, difficulties met)
	Opinion and views on the organisation and the management of the experimental protocol
	Relationships with other stakeholders
**Motivation and interest behind participation**	Factors that influenced participation
	Personal interest
	Third-party opinions or arguments that influenced the decision
	Changes in viewpoint
**Drawbacks and obstacles to participation**	Factors that influenced refusal to participate
	Reasons for dissatisfaction
	Third-party opinions or arguments that influenced the decision
	Changes in viewpoint
**Knowledge of the gamma-interferon test**	Ability to talk about the test and present it
	First impressions of it (drawbacks, advantages, comparisons with other tests)
	Changes in viewpoint
	Issues on its use for screening bovine tuberculosis
**Issues regarding changes to the current control**	Opinion about the management procedure for suspect bovine tuberculosis cases
	Opinion and feelings on the lock-up period
	Issues at stake for shortening the lock-up period
**Concluding remarks**	General opinion on the trial
	Please list five adjectives to describe the experimental protocol or the gamma-interferon test?
	Ways to improve the trial
	Others remarks

In-depth interviews were conducted at the participant’s location of choice: most often at their home for farmers, at their veterinary practice for veterinarians, and at their office for other participants. Although, ideally, interviews should have been conducted individually without witnesses to facilitate expression of personal opinions [18], one interview was conducted with the farmer and his spouse, upon the farmer’s request. All interviews were conducted face to face by the same person (first author) to encourage responses and to ensure comparability of collected information [14]. At the time of the study, the first author was preparing for a Master’s degree in management, social and human sciences, and she introduced herself as a student in the humanities.

At the beginning of the interview, the aim and background of the study were explained, as well as the confidentiality of the opinions expressed. Although the interviewer used guides, participants were free–and encouraged–to elaborate or introduce any other information that they felt was relevant. All interviews were recorded using a digital audio recorder to aid and facilitate analysis and to avoid over-structuring the discussion. However, as expected, most participants gave further information or details at the end of the interview, once the recorder had been turned off. The interviewer noted these last pieces of data and field notes were reviewed after each interview.

Data collection continued until saturation of ideas occurred. For every type of stakeholder, each eligible and pre-selected participant was interviewed, except for farmers. Twenty four semi-structured interviews were performed in Ardennes from April to May 2015 (Table 2). Interviews lasted between 27 to 77 minutes (not counting discussions after interview recording). Among the 11 farmers interviewed, 8 took part in the EP during the first campaign (2013–2014) and 3 during the second (2014–2015). Moreover, among the eight farmers taking part in the first campaign, four farmers were eligible to participate in the EP again during the second campaign, but all declined.

**Table 2:** Characteristics of interviews.

Typology	Number of interviewees	Average length of interview
**Farmers**	11	55 minutes
**Veterinarians**	5	54 minutes
**GTV representatives**	2	71 minutes
**GDS representatives**	2	45 minutes
**Veterinary services agents**	3	61 minutes
**Departmental testing laboratory agent**	1	75 minutes

### Data analysis

The *verbatim* interview recordings were manually transcribed, compiled with field notes and log book, and then analysed thematically [22]. The first step in data analysis involved reading through all of the transcripts to get a sense of the data set as a whole. A thematic analysis [23] was then performed on the transcripts, following the methodology outlined by Beaud and Weber [14]. The data were analysed using the constant comparative method of qualitative data analysis [24]. They were examined with regard to the research questions, significant text fragments were identified, and initial codes (basic units of analysis whose central meaning is described in a short statement) were established on concepts. Fragments were grouped into categories, i.e. groups of content that share common features. Similarly, categories were organised around themes. These themes (Table 3) were created to link underlying meanings that reoccurred within categories [25, 26].

**Table 3:** Overview of the research aims linked to the themes and categories that emerged during data analysis.

Research aim
Theme
Category
Advance understanding of the factors that influence farmer participation in the experimental protocol to assess gamma-interferon testing for the diagnosis of bovine tuberculosis
Key to participation and stakeholder influences
Factors that encourage farmer participation
Stakeholder influence: role of veterinarian
Insufficient knowledge of the experimental protocol and its aims
Elements behind refusal to participate
Reversal of the trend: from refusal to systematic rejection
Describe the perception of gamma-interferon testing and its issues
Perception of the new test
Participants’ opinions on the test
Opinions on the influence of the test on farmer participation
Shorten lock-up period
Economic impacts on farmers
Psychological impacts on farmers
The request for a new test to replace the skin test in live animals
Farmers made no link between this stake and the use of gamma-interferon testing
Provide recommendations for designing trials so that they promote farmer participation or animal health stakeholder participation in general
Understanding refusals to avoid them
From refusal to systematic rejection
Understanding the lack of knowledge on the EP
How to counter failure
Elements that promote farmer participation
Veterinarians play a crucial albeit difficult role

The analysis was conducted using a circular process: repetitions of forward and backward movements from transcripts, gathered text fragments, codes and introduction of inference [27, 28]. Before making any inference, evidence to the contrary was searched for and speechlessness was prospected. Triangulation and iteration principles were strictly applied, involving the search for repeats and synergy in transcripts and cross-checking of the information given by at least three stakeholders of different types (Table 2) to validate the inference [22, 29]. These steps in the analysis fulfilled the expectations of trustworthiness and rigour [27, 28, 30, 31].

All data presented in the results section reflect the observations, insights, and opinions expressed by interview respondents [32]. To protect participant confidentiality, their identities, ages and herd size (for farmers) are not detailed in this paper, although the typology of stakeholders is linked to citation. Note that all *verbatim* quotes cited in this paper have been translated from French (S2, S3 and S4 Tables).

## Results

### First aim: Understanding the factors that influence farmers to take part in the experimental protocol

#### Factors that encouraged farmer participation

One theme and five categories linked to the aim of advancing understanding of the factors that influence farmers emerged from the data analyses. Different reasons and stakeholder influences (role of the veterinarian) were identified to explain the farmers’ choices; they are listed in Table 4 and linked with their level of reality. No qualitative differences were highlighted between factors mentioned by farmers who participated in the EP during the first campaign (2013–2014) and those who participated in the second (2014–2015). Nevertheless, the change in the detection threshold of the IFN test was an additional argument only mentioned by farmers in the second campaign, as it had changed between the two campaigns.

**Table 4:** Factors explaining farmer’ participation in the EP with respect to their level of reality.

Factor promoting participation	Reality
Hope to maintain herd health status	**Optional fact depending on the level of the suspicion (strong or weak)**
Hope to avoid slaughter for *post-mortem* confirmation of the infection	
Trust in veterinarian’s proposition	**Relationship perception**
Assumption that management of suspect cases will be more generous and shorter	**Assumption due to misunderstandings or lack of knowledge on the experimental trial**
Assumption that gamma-interferon testing is more reliable than the skin test	
Assumption that gamma-interferon testing is unequivocal for suspect cases	
Assumption that total culling of a herd in case of real infection depends on farmer participation in the EP	
The change in detection threshold	**Existing situation**
Participation in the interests of contributing to scientific progress	

By participating, farmers expressed two kinds of hopes: *(i)* the hope of preserving their qualification (animal health status) that allowed them to continue to sell their products in France despite the suspicion of bTB, and *(ii)* the hope of avoiding slaughter of cattle for *post-mortem* confirmation of the infection if the IFN test was negative.

Most of the farmers participated in the EP especially for the assumed quality of the IFN test. Some farmers imagined that this new test could categorically confirm or rule out infection. Some thought that the lock-up period would be shortened in the EP compared with the standard protocol. For some farmers, these preconceptions came from the knowledge they had on the IFN test: they knew it had been used in other departments of France and that it was used in partial culling (but none knew this use was in parallel with ST). They therefore assumed that the IFN test was reliable. Otherwise, several farmers explained that they thought they could avoid total culling of the herd in the case of proven infection because they had taken part in the EP.

During the interviews, farmers almost always referred to their veterinarians in their arguments to participate in the EP. Nevertheless, they always noted that the first contacts they discussed the EP with were veterinary services agents. On the other hand, some farmers explained their participation in the EP only because their veterinarian told them about it, without remembering clearly the arguments their veterinarian had used to convince them. This analysis starkly highlighted the confidence that most farmers (10/11) have in their veterinarian: they considered their veterinarians as an adviser for the improvement of the state of health of livestock. As a consequence, they took into consideration what their veterinarians proposed. One of them explained: “*I could have said ‘no’ [meaning to the EP]… the final decision was mine*. *I chose to follow their advice”*. The relationship between farmers and veterinarians seems to have played a major role in the decision-making process.

Only two farmers mentioned they participated in the EP to help science and to drive progress by evaluating the IFN test. However, this reason was never formulated alone, suggesting that scientific progress was not sufficient for farmers. However, it clearly appeared that shortening the lock-up period with the use of the IFN test was a real incentive for them (*theme 2*).

#### Insufficient knowledge of the experimental protocol and its aims

Among farmers, only a few respondents (2/11) were aware that the EP was a temporary animal health trial to assess the IFN test. Most farmers thought the EP would give them more flexibility if suspect cases were revealed and that the lock-up period would be shortened. Thus, these farmers confused the long-term objectives of the EP (recognition of the IFN test for confirming or ruling out suspected cases in Europe, which would shorten the lock-up period) and the short-term objectives of the EP that justified its implementation (experimental assessment of the IFN test to determine whether it could replace the SST carried out 42 days after the ST). Some farmers took the use of the IFN test for granted and thought the EP was a new flexible way of managing suspect cases. This confusion was originally the source of many misunderstandings and assumptions. This analysis highlighted the discrepancy between the importance of shortening the lock-up period and the participation criteria, which were based on other arguments.

After the first non-negative ST screening results (suspect case), participation in the EP was proposed to the farmers both by their veterinarians and by veterinary agents who contacted them by telephone. At the same time, an enrolment form was sent to farmers. Veterinary agents were knowledgeable about both the outcomes of the EP and most of the veterinarians interviewed knew the aims of the trial. However, when they explained it to the farmers, they focused on the long-term objectives and the information given may have been partial or approximated. For instance, one veterinarian stated that he explained the EP with the following words “*the aim is to facilitate tuberculosis detection and management*”. Explanations like these likely confused farmers. This analysis clearly highlighted the complexity of the EP and the difficulty in communicating it clearly.

#### Elements of refusal

Even though most farmers could continue selling their products during the lock-up period in the EP, they were not able to avoid diagnostic slaughter (DS) of suspect cattle (slaughter for *post-mortem* confirmation of the infection). Based on the testimonies gathered, DS was the main element that led farmers to turn down the proposal to take part in the EP (this argument was cited by all stakeholders interviewed). DS was always an unpleasant experience for the farmers who participated in the EP because they thought that their participation was a way of avoiding this process. Many farmers and veterinarians did not realise that they might need to slaughter suspect animals, even if SST results were negative, because DS was based on ST and IFN results. Consequently, several farmers (7/11) and veterinarians (5/7, including GTV representatives) summed up the EP with the following words: “*If you want to cull cattle*, *go ahead and join*!*”* or “*I have the feeling that to slaughter animals you just need to do the interferon test*”. Almost all farmers interviewed agreed that they would have preferred their suspect animals be slaughtered after the first non-negative ST, instead of taking part in the EP. This analysis highlighted that participation in the EP was not experienced as a fully satisfactory alternative. Furthermore, farmers developed a negative perception of the IFN test, even though the assessment of this test was still in progress. After culling of suspect animals that had non-negative IFN results, the *post-mortem* diagnosis usually overturned the suspicion. The pathology of bTB is complex and animals may be infected even when the *post-mortem* diagnosis based on bacterial cultures is unable to confirm it. However, farmers nonetheless considered straightaway that the IFN test was unreliable. Furthermore, some farmers discovered after their participation in the EP that the IFN test can yield inconclusive results, even though they thought this test could have only two modalities (positive or negative). This contributed to increasing their dissatisfaction with the EP.

Our analysis showed that stakeholder influences took place around the EP and demonstrated how they affected farmer participation. At first, veterinarians viewed the EP favourably, but they stopped supporting it during the second campaign for two main reasons: *(i)* too many DSs occurred following results of SST and IFN tests, and *(ii)* they considered that the IFN test was not more reliable or more effective than the SST. For instance, one farmer explained “*veterinarians told us not to do it [the IFN test] because it wasn’t reliable*”. Across the board, the analysis demonstrated that the refusal was partly due to the veterinarian’s position and ethical views: the veterinarians interviewed could not afford to propose the EP if diagnostic slaughters were more frequent than in the standard protocol.

#### Reversal of the trend: From refusal to systematic rejection

Because the farmers who participated in the EP hoped to avoid the DS and expected to shorten management time of suspected cases, the real constraints were exacerbated by the misunderstanding of what happened. One laboratory agent interviewed explained: “*In the end*, *farmers are extremely frustrated because they thought it would improve their situation*”. This frustration echoed their own opposition. In this case, opposition was the duality between a future expected to be better and a different reality. This feeling was assessed and recognised during most of the interviews with farmers (9/11). It was usually concomitant with the expression of dissatisfaction. These disappointments may have contributed to the systematic rejection of the EP by some stakeholders, especially farmers and veterinarians; this hypothesis was validated for at least 9 farmers interviewed out of 11. Otherwise, four farmers could have participated in the EP again during the second campaign, but all of them declined because they did not want “*to hear about it anymore*”, to repeat the expression used by one of them. This rejection was identified from interviews with veterinarians as well (5/7, including GTV representatives).

**Fig 3:**
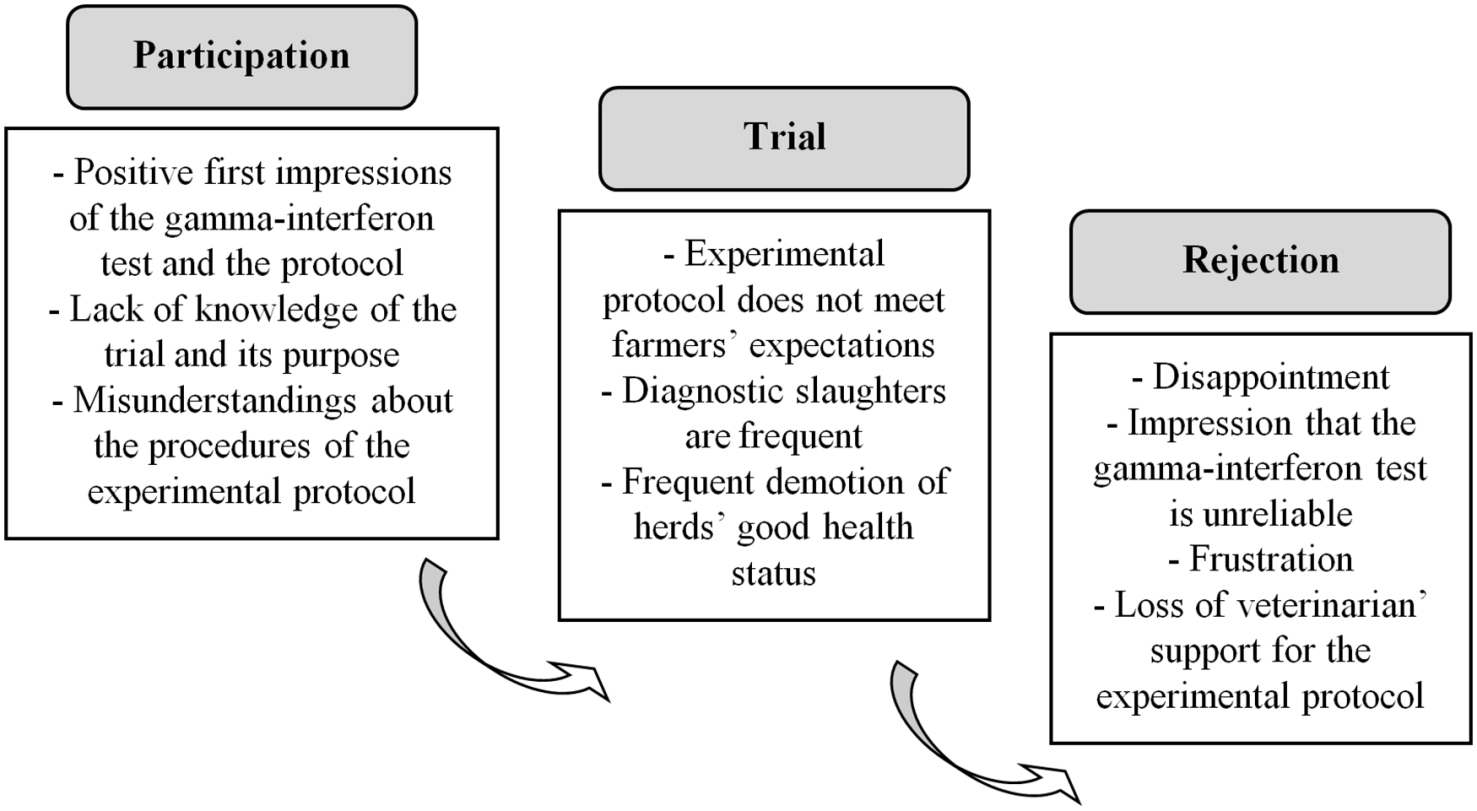
Explanatory diagram of the farmers’ participation and their rejection of the EP.

To sum up, misunderstandings and misconceptions about the IFN test, and the confusion between the long-term and short-term objectives of the EP led not only to the refusal to join the EP (farmers turned down the offer), but also to broad rejection of the trial (clear and systematic crowding-out). Moreover, this position resulted in conveying a negative image of the IFN test and promoted the rejection of the EP for the other stakeholders potentially involved. Even the farmer who had avoided diagnostic slaughter of cattle thanks to the EP did not want to participate again the following year. Fig 3 shows a general picture of the situation.

### Second aim: Perception of the gamma-interferon test and its stake

#### Perception of the trial and the IFN test

At the end of the interview, respondents were asked to give five adjectives to describe their feelings and opinions about the EP or the IFN test. All respondents listed adjectives: 39 different adjectives were cited to describe the EP (Table 5) and 30 different adjectives were formulated to characterise the IFN test (Table 6).

**Table 5:** Adjectives chosen by stakeholders to characterise the EP, regarding the aspect described.

Aspect	Adjectives cited by farmers	Adjectives cited by other stakeholders
**Practical implementation**	*Unsuitable*, *long*, *cumbersome*, *rigid*, *unrealistic*, *poorly initiated*	*Unadapted*, *time-consuming*, *slow*, *too heavy*, *perfectible*, *not easy to understand*, *demanding*, *experimental*
**Operating process**	*Strict*, *complicated*, *unclear*, *unconvincing*, *worrying*	*Strict*, *complicated*, *worrying*, *very technical*, *inconclusive*, *obscure*, *confusing*, *vague*, *lousy*, *improvable*
**Consequences and feelings**	*Interfering*, *punitive*, *strict*, *upsetting*	*Risky*, *not easily acceptable*, *rejected*, *inadvisable*, *stressful*, *unilateral*^1^
Financial aspect	*Costly*, *expensive*	*Costly*

^1^- no feedback given to participant

**Table 6:** Adjectives chosen by stakeholders to characterise the IFN test according to connotations.

Stakeholder	Adjectives with a positive connotation	Adjectives with a negative connotation
Farmers	*Safe*^1^, *fast*, *simple*, *accurate*, *new*, *convenient*, *bearable*	*Expensive*, *costly*, *inconvenient*^3^, *ineffective*, *strict*^3^, *complicated*, *bad*^4^, *doubtful*, *approximate*, *uncertain*, *not really reliable*, *unreliable*, *unpredictable*, *useless*, *improvable*, *unsuitable*
Other stakeholders	*Easy*, *safe*^1^, *fast*, *simple*, *convenient*, *objective*^2^, *interesting*, *beneficial*	*Expensive*, *costly*, *ineffective*, *strict*^3^, *arbitrary*, *not really reliable*, *unreliable*, *confusing*, *mixed*^4^, *useless*, *adaptable*

^1^- Related to the operating method

^2^- Related to the formulation of the outcomes, in comparison with the skin test and results formulated by the veterinarian

^3^- Related to the shipment of samples to the departmental testing laboratory

^4^- Related to the three outcomes of the test: positive, negative and inconclusive

First, most of the adjectives chosen to describe the EP had negative connotations. Only three adjectives had neutral or positive connotations: “*improvable*”, “*perfectible*”, and “*experimental*”, but these adjectives were not given by farmers. Concerning the practical implementation of the EP, adjectives illustrated various aspects. Terms such as “*unsuitable*”, “*unadapted*” and “*unrealistic*” were recurrent. Other adjectives described the commitment and involvement: “*demanding*”, “*too heavy*”, and “*time-consuming*”. Focusing on adjectives chosen by farmers to characterise the consequences and their final opinion on the EP, we noticed that words were usually linked to the concept of constraints (“*interfering*”, “*punitive*”) and illustrated how these constraints had affected the participation in the EP (development of rejection) or farmers’ feelings (development of stress, for example). This perception had to be considered in conjunction with the complexity of the EP, the short sample delivery time (to carry out the IFN test on time), and the technical characteristics of Ardennes, which has a departmental laboratory that could only perform one step of the IFN analysis. All stakeholders interviewed expressed the same view: the technical procedures of the EP (meaning the management of the samples, their planning, their treatment and analysis) were quite cumbersome and strict. However, the organisation and management of these technical procedures (which were determined by the active commitment of participants and coordinated by veterinary services agents) appeared efficient and were not denounced by stakeholders.

Although not quantitative, the analysis of most of the adjectives chosen to describe the IFN test showed that there were negative connotations (19 different adjectives related to 39 quotes), but adjectives with a positive meaning were relatively frequent (11 different adjectives related to 23 quotes). Most of the time, the negative adjectives referred to the interpretation of the test, whereas positively connoted adjectives referred to the technical characteristics of the test (blood sample) and were often compared with those of the ST, which was considered subjective, time-consuming and tedious. Furthermore, focusing on the lexical fields, we noticed that adjectives linked to the interpretation of the test (16 quotes of 62) referred to lexical fields of approximation and confusion. These adjectives were formulated by farmers and veterinarians and they highlighted the opinion of the stakeholders about the IFN test and its interpretation: they thought it was unreliable. In addition, it is worth mentioning that this negative view and its generalisation constituted one factor leading farmers to refuse to join the EP during the second campaign. Paradoxically, despite the fact that the IFN test is more expensive than the ST, the lexical field of cost was neither recurrent nor predominant (two different adjectives related to six quotes). This statement should, however, be put into perspective with the procedures of the trial: the administrative authority (French Directorate General for Food) was responsible for financing the EP and veterinary services agents interviewed were a minority in our study.

#### Shorten lock-up period: A real incentive

Because of its economic impacts on farmers, the lock-up period of six weeks due to a suspected case constituted a highly unsatisfactory component of bTB management for all stakeholders. The lock-up period is particularly restrictive if it occurs when the herd is supposed to turn out to pasture (because farmers have to keep feeding cattle in stalls), during the high season for selling young calves and beef steers, and during beef cattle competitions or competitive dairy competitions (negative valuation effect on herds). Moreover, even if farmers are allowed to send their cattle to slaughter during the lock-up period after receiving a special permit issued by the veterinary services, animals are devalued and farmers suffer indirect financial losses. All stakeholders interviewed strongly regretted this arbitrary process of devaluation followed by slaughterhouses.

The analysis of data clearly revealed that the lock-up period has a psychological component. Farmers agreed that the waiting period is especially fraught with anxiety and 3 respondents out of 11 described unbearable waiting periods. Respondents other than farmers expressed and emphasised obvious concerns about this particular point. In case of suspicion, herds are locked up and farmers have to wait six weeks without knowing what will happen subsequently: they may earn back their health status (if the suspicion is disproved by the SST) or they may have to cull a certain number of suspect animals for *post-mortem* investigation (if the SST is non-negative). Finally, there is always the risk that the suspect animals are indeed infected and that the whole herd will be slaughtered, annihilating the farmer’s work and efforts. To further illustrate the psychological dimension, one veterinarian reported that farmers refused to do screening in December, because they were concerned about the emotional consequences of a lock-up period during the Christmas and New Year’s celebrations.

Furthermore, respondents criticised the ST, which still represents the only recognised method in European Union regulations to diagnose bTB in cattle. They found the ST strict, time-consuming (veterinarians have to visit the day of the injection and for the skin examination three days later), and even dangerous (injection in the neck, not far from the horns). The skin thickness was usually measured with vernier calipers, but in some departments operators are allowed to check the skin by hand, without using the special device. This difference in ST practices sparked comments on the lack of national harmonisation on caliper use. Moreover, the subjectivity of the test was strongly criticised by farmers and all veterinarians. One of them explained: *“For me*, *skin tests are really biased*, *because it is a matter of [just a few] millimetres with the caliper*. *Afterwards*, *when they [cows] are well tied-up*, *it’s OK*, *but when they go through the containment corridor and they are stressed… And it depends on the fold of skin we choose*: *it’s biased*, *it’s really biased I think”*.

Shortening the lock-up period appeared to be a genuine issue for all stakeholders due to its economic and psychological impacts on farmers, and they expressed the need to develop a new test to screen for bTB in cattle. However, these statements did not strictly concur with the factors that explained why farmers joined the EP. In fact, this may explain why they had a good initial opinion of the IFN test, but scientific progress and the recognition of a new test were not key determinants in the participation of farmers in the EP. This discrepancy is explained in part by their misunderstanding and their lack of knowledge about the EP.

## Discussion

### Specificity of the qualitative approach

Our study followed a qualitative comprehensive sociological approach as endorsed by Max Weber [17]. A qualitative approach is a valuable way of understanding the diversity and extent of opinions, and it leads to deeper understanding of the roles that circumstances, motivations, relationships and context play in human behaviour [17,33]. Although this approach does not lend itself to statistical inference, it helps to understand why reactions occurred [11, 27, 32]. The qualitative approach is therefore well suited to gain insight into human decisions (24) and to meet our goals. Our purpose was to enhance understanding and broaden awareness of farmer participation in animal health trials. Furthermore, our study investigated possible solutions or incentives to encourage farmer participation.

To our knowledge, this paper is the first to explore cattle farmers’ motivations to join a national trial and their opinion about changes in the way suspected bTB cases are managed. Each opinion is qualitatively important and helps to understand the decision-making process [17]. Although we cannot strictly quantify the factors behind motivation and views, nor can we be confident that we have captured every opinion held [34], we consider that our approach highlighted strong convergences within a given theme and captured novel information that cannot be obtained using a quantitative questionnaire methodology.

The confidentiality of the interviews ensured the trustworthiness of participant answers [28, 35]. By following analysis and sampling rules (triangulation, iteration and saturation) [29, 30], we can validate our results at the departmental level. To further generalise our conclusions, we have to first consider the specificities of the department chosen for the study. Only a few outbreaks of bTB are detected each year in Ardennes (around five), but in other departments with higher prevalence such as Côte-d’Or, the EP was mandatory (enforced through a prefectural ordinance). In Ardennes, the departmental analysis laboratory was not accredited to carry out the whole IFN analysis, only the first step. Additional technical limitations may have complicated the EP and influenced stakeholder views. Moreover, when they had to slaughter suspected animals for *post-mortem* diagnosis, most farmers interviewed reported that they had to cull animals with high breeding or genetic value. We can logically assume that the psychological consequences of diagnostic slaughter were amplified. On the other hand, there was no tension between veterinarians and farmers due to the enforced prophylactic measures. However, in some departments, the situation is quite different [36], so we can expect that in these departments the relationship between farmers and veterinarians had a lower impact on farmer participation. Nevertheless, based on all these considerations, we consider our approach robust and reliable. Therefore, further ideas for recommendations can be gleaned or generalised.

### Formulation of recommendations

The commitment and support of targeted stakeholders for a change (here the EP) depends on how well the stakeholders understand the objective and procedures behind the change, and commitment is particularly closely related to the personal interpretation they have of the change or their interest in it [37–40]. Our study investigated key components and incentives to manage the design of a participatory animal health trial and how to encourage farmer participation. Our findings may provide further insights into new ways of setting up a trial, or improving communication on the trial to facilitate and thus increase voluntary farmer participation and avoid negative consequences of participation based on assumptions. Although this study was conducted for an animal health trial in one specific country, the underlying principles and recommendation drawn from it are applicable to other trials involving researchers, animal health managers and field participants.

#### Understanding the lack of knowledge on the EP

Our analysis demonstrated that stakeholders varied in how well they understood the aims of the EP. Several explanatory hypotheses can be tested to explain these misunderstandings and this lack of knowledge, which had drastic consequences. Firstly, an analysis of French trade magazines (targeting farmers and veterinarians), published between September 2013 and July 2015, revealed only two articles that addressed the EP and, furthermore, only succinctly described the aims of the EP [36]. Thus, sources of information on the EP were scarce other than the memorandum from the French Directorate General for Food sent to veterinarians and the enrolment form given to farmers.

Secondly, the EP was a particularly complex trial (Fig 1): the decision scheme applied to cattle varied with the level of bTB suspicion and the results of the ST, SST and IFN tests (three possible outcomes). In addition, the long-term and short-term aims of the EP were easily conflated and led to confusion. Before examining difficulties in interpretation of the EP, it is important to understand that bTB is a complex disease due to its pathology, its chronicity [1], its epidemiology [38], and the lack of a completely sensitive and specific test to diagnose live cattle [3, 7, 39]. Moreover, veterinarians face difficulties in the implementation of tests due to inadequate on-farm testing conditions or deficiencies in the development or supervision of testing skills among trainees and newly qualified veterinarians [40]. Deviations from testing procedures have in fact been identified [39]. The subsequent disease control and prevention procedures are difficult to comprehend and to explain. For non-scientists, the concept of disease causation, incubation and diagnosis are difficult to understand [41]. In addition, the low predictive value of non-negative results due to the low prevalence of bTB and cross-reactions with non-pathogenic mycobacteria lead to false-positive results [5]. Thus two diagnostic tests can give contradictory results [7], which clearly demotivated veterinarians and farmers.

Our data analysis revealed how communication about bovine tuberculosis can be difficult. This suggests there is a need to provide communication tools to veterinarians to face this challenge. One veterinarian interviewed criticised the current situation because this disease is almost mysterious, owing to the imperfections of the test and the need to carry out two tests associated in series to confirm suspected cases; he said: *“We can’t understand everything*! *We [meaning veterinarians] are on the side of the scientists and we have to explain to people […] that we are dealing with one of the rare diseases where the diagnosis is a nightmare*! *That’s the problem*: *the diagnosis is a nightmare*! *[Exasperated tone]”*. In this context, providing clear and accurate explanations of the EP was difficult, particularly for veterinary services officers who called farmers by telephone without having pre-determined communication tools. Testimonies collected from farmers, veterinarians and the laboratory technician confirmed this hypothesis. One veterinary services agent reported, *“We spent a long time on the phone with farmers*, *but the problem is that the protocol [the EP] is quite complicated*, *let alone explaining it on the phone… [Raised eyebrows]”*. Comments from all stakeholders involved concurred that the EP was a complicated animal health trial that was difficult to understand and explain.

Moreover, during the interviews, many stakeholders came up against the term “*experimental protocol*”. In fact, stakeholders usually used the word “*interferon*” to both talk about the experimental protocol and the gamma-interferon test. We can expect that the use of an abbreviation contributed to obscuring the fact that it was a temporary experimental trial.

#### How to counter the failure

The analysis of factors behind the successes and failures, demonstrates the importance of communicating and providing clear and accurate information and explanations to encourage stakeholders and actors to commit to a change in practices [8,42], and in our particular case to participate in the EP. In addition, one strategy to overcome resistance to change is to focus on communication (information, discussion and participation) to avoid actors’ misunderstanding [43–45]. Generally, communication on infectious diseases, their prevention and control requires special language and adapted channels [41,46]. This paper advocates the value of providing timely, adapted and succinct descriptions of the objectives and process of formal field research activities to farmers who will potentially participate.

Alders et Bagnol (2007) define effective communication material as having five characteristics: clear, consistent, credible, practical and correct [46]. From our findings, the clarity of the information was essential and two supplementary criteria could be added: appropriateness and comprehensiveness. To present the EP to farmers (whose herds had non-negative test results), veterinary services agents sent them an enrolment form by post. This form consisted in two typed pages [6] and was originally published by the French Directorate General for Food. Then, using the form, an officer called the farmer, explained the EP and answered any questions. As a result, an individualised method of communication was used to present the EP to farmers. The analysis of data demonstrated that confusion and misunderstandings about the EP were partly attributable to this enrolment form, even though veterinary officers had verbally presented it. Combining the interviews with farmers and veterinarians showed that the explanatory text was too long and inappropriate, e.g. “*The enrolment form is too wordy*” or “*It’s too vague*, *there is too much text*”. Furthermore, the objectives of the EP are mentioned in two different paragraphs, perhaps contributing to confusion. To make it clearer, aims should be explained in only one paragraph dedicated to this purpose, clearly distinguishing the long-term and short-term goals. Revising the objectives would ensure good general knowledge of the pros and cons of the EP.

The communication material used was not comprehensive, leading to resistance to change because actors gradually lose confidence in the trial [43,44]. Stakeholders regretted the lack of precision and information on the consequences of committing to the EP. On the form, it was indicated that: “*In this protocol*, *you can benefit from relief measures with regard to the demotion of the good health status qualification*, *and you can benefit from more targeted measures regarding the slaughter of suspect animals*”. These elements were true, but the advantages depended highly on the level of the suspicion and the results from the ST and IFN tests. The form’s sentence suggested that culling suspect cattle would be less common, but in practice this was not the case. However, in general, the EP measures were stricter than the normal measures required in suspected case management (Fig 1). In addition, those involved may have focused at first on the absence of withdrawal of the farm’s good health status (i.e. movements of animals and animal products would continue to be authorised). However, in practice, there was little advantage related to the EP: the IFN test had to be performed twice (after the first ST and six weeks later with SST), even if a diagnostic cull had already been scheduled subsequently.

In addition, all farmers argued that the enrolment form was not adapted to them and that it did not provide a clear and complete picture of the EP and its consequences. The form’s flaws subsequently had serious repercussions, generating misunderstandings, incomprehension, a sense of frustration and even discontent. Enticott (2012) reports that protocols can take many forms (written instructions, diagrams, flow charts or algorithms) to guide professionals in a sequence of steps [39]. From farmers’ comments, the first recommendation for improvement involves supplementing the current text with labelled drawings or diagrams. In general, based on this example, farmers are more receptive to diagrams than to text, and clear visuals should be provided when setting up a participatory trial. A previous study conducted in Myanmar highlighted the importance of clear illustrations to sensitise farmers to research findings [41]. In Sudan, rinderpest control proved very successful thanks to the development of stories, songs and poems to increase awareness among communities [47].

Explanations of the institutional and operational procedures were crucial for handling the EP and its procedures and objective. This type of information prevents hasty and erroneous deductions [8]. However, erroneous conclusions were reached regarding the EP, sometimes causing confusion [43]. We therefore analysed the feelings of stakeholders (other than farmers) on the information they received from the French Directorate General for Food who planned the EP. We must consider the ability of the stakeholders to explain and provide details on the EP in regard to the general level of information they receive. The EP was described in two memorandums No. 2013–8162 and No. 214–864 [6,10], the second one updating the first, but without major changes. Four types of stakeholders read these memorandums: veterinary services agents, GDS representatives, GTV representatives, and laboratory technicians. First of all, we considered that information retained by stakeholders depended largely on the data provided in the documents (the two memorandums), in both quantitative terms (exhaustiveness and completeness) and qualitative ones (accurateness, homogeneity and clarity). From a qualitative point of view, all stakeholders (excluding farmers) reported that the memorandums were not easy to understand and some parts were not very clear. From a quantitative point of view, most regretted that documents were not sufficient to understand all the terms and conditions of the EP. Veterinary officers reported that they needed to contact a regional coordinator of the EP (appointed by the Directorate General for Food) to clarify the conditions of the EP. Veterinary officers, GDS representatives and veterinarians expressed the wish to have details and further explanations of the IFN test, its intrinsic characteristics, and its advantages over the skin tests. Lastly, some of them regretted the absence of communication tools about the EP and the IFN test, which could be used to inform farmers.

Ultimately, there is a clear need to involve farmers and veterinarians in the early stages of trials in order to identify potential obstacles and ways of overcoming them. In the literature, one popular strategy for dealing with resistance to change is to get the people involved to “participate” in making the change [43, 45, 48]. The participatory process provides comprehensive learning for stakeholders involved and takes into account scientific results and the participants’ own knowledge and experience [49]. Analogously, participatory epidemiology is defined by Catley *et al*. (2012) as “the systematic use of participatory approaches and methods to improve understanding of diseases and options for animal disease control” [50]. Participatory methods were used for the development of an antimicrobial stewardship policy in the UK, involving intensive collaboration and dialogue between dairy producers, veterinarians, scientists and industry partners [51]. This cooperation led to the development of credible and practical recommendations designed to deliver real on-farm changes in the use of antibiotics. Similarly, in developing countries [52] governments have initiated and co-ordinated control programmes with farmers to cope with various diseases, rinderpest in Sudan [47], tick born disease in Zimbabwe [53], African swine fever along the Kenya-Uganda border [54] or highly pathogenic avian influenza in Indonesia [55]. The success of these programmes is linked to the involvement of farmers in the design, implementation and evaluation of them. Considering these successful examples, further recommendations for animal health trials can be formulated: the use of participatory methods will be useful to involve farmers in all stages of the research, from design to implementation, rather than being asked to join or even coerced into joining a final project. Participatory methods can also contribute to identifying efficient means of communication [47]. Thus, workshops [56] with farmers, veterinarians and veterinary agents will help to explain both bTB and complex technical issues related to the trial. Moreover, our findings give some support to the view that participatory approaches could play a role in developed countries [50] to ensure that farmers and researchers understand their respective perspectives, objectives and priorities.

In the light of this analysis, it appears necessary to complete the memorandum with scientific information and to clarify the procedures involved in the management of suspected cases. This would constitute one strategy to overcome resistance to change [44, 56]. Overall, three recommendations can be formulated for setting up a trial: *(i)* include stakeholders at early stages of research design through the use of workshops or participatory methods, *(ii)* prepare tools to explain the trial to facilitate communication between stakeholders with different backgrounds (veterinary services agents and farmers, for instance), and *(iii)* publish detailed communication material with scientific explanations on the tests and the consequences of the trial. Although lack of clear communication was a major failure for the EP, communication can also be considered as a main lever for action, as a way of exerting positive improvement to enhance farmer participation [41, 47].

Kotter (1995) emphasised that communication was important at the different steps of the change process to maintain stakeholders aware of the situation and the results achieved [56]. In the course of the interviews or before the interviewer left, most of the stakeholders asked for news on the EP and inquired about what was going to happen next. Moreover, all respondents expressed the wish to receive feedback on it, in particular as a report or a short note. This request appeared legitimate, especially for farmers who accepted the strict conditions associated with their participation in the EP. Communicating on the progress of the EP addresses various strategic issues. Firstly, sending reports informs the stakeholders regularly and provides them with a general overview of the experimental system [8]. Secondly, producing a comprehensive updated report helps to enhance the value of the work and involvement of the stakeholders, thereby acknowledging their participation [56]. It is important that the results of the trial be accessible to all involved stakeholders because this helps to justify their efforts [37]. Finally, as a communication tool, this kind of report creates connections between stakeholders [9].

In addition, if the IFN test proves to be useful in detecting bTB after the first ST in the future, it would be beneficial to adjust the current names of the test’s outcomes. Currently, three outcomes are distinguished: negative, positive and inconclusive. An inconclusive result is obtained when the different ratios calculated from optical density do not concur (the IFN test is partly based on an enzyme-linked immunosorbent assay). Based on comments, the term “*inconclusive*” was repeatedly criticised by stakeholders. Because the concept of “*inconclusive*” had not been presented nor explained, it was particularly difficult for farmers to comprehend. Furthermore, some farmers and veterinarians took “*inconclusive*” to mean “*uninterpretable*”, making it seem unacceptable that suspect animals be culled due to an inconclusive result. One veterinarian explained: *“It’s an outcome we don’t want to stick our necks out for*! *[Forced laughter] No*, *that’s it*, *one test reacts and another doesn’t*, *we don’t know*. *And even for us it’s difficult to explain it simply to farmers”*. Another summed up the situation as follows: “*It’s hard to understand*, *it’s difficult to accept”*. One veterinary agent thought it would have been preferable to use a scale; he explained, “*Maybe we should use levels*, *for instance […] a green/orange/red level*. *Perhaps because an inconclusive outcome means there is no conclusion*. *And people*, *including me at the very beginning*, *thought it meant uninterpretable*. *But here [with a colour scale]*, *we see it as a doubtful result*: *one thing has been modified*, *the other thing didn’t change”*. Perhaps if this term had been more understandable, the diagnostic slaughter based on inconclusive results may have been better tolerated, or at least better understood.

#### Veterinarians play a crucial albeit difficult role

Cattle farmers considered their veterinarian in different ways: *(i)* as a partner who works to improve cattle farming, *(ii)* a doctor who cures and treats animals and the herd, and *(iii)* an adviser who provides scientific information on the technical problems encountered (zootechnical information, nutrition, etc.), and sometimes even as a friend when a rapport develops [57–59]. In the literature, authors agree that the veterinarian is one of the major sources of information for farmers and our results highlighted this conclusion [59–62]. Nearly every farmer and veterinarian interviewed attested to the role of the veterinarian (far from the limited role of “firefighter”) and to the special nature of the relationship between them, which facilitated effective transmission of information. Compiling comments showed that during an animal health trial such as the EP, the veterinarian is the most appropriate person to relay the information to farmers in the field. He serves as an interface between farmers and science [39, 41, 59], and our analysis matched with this overarching impression. Therefore, efforts to keep veterinarians informed of bTB testing procedures [63] and test characteristics are particularly important to ensure they understand the issues linked to the screening and are able to make an informed judgment of the likelihood of the disease [39].

All veterinarians confirmed the view that they preferred to preserve their relationship with their farmer instead of strictly encouraging participation in the EP for a laudable scientific purpose. Some veterinarians explained that veterinary services cannot demand everything of veterinarians, particularly when the requirement appears to threaten their relationship with farmers. In the particular case of this trial, veterinary agents recommended that they briefly present the EP to their farmers when cattle showed non-negative results to the first ST screening. One veterinarian summed up: “*For us*, *the interest of farmers comes first*”. Thus, if farmers expressed strong criticisms or concerns about one trial that was presented or advised by their veterinarians, veterinarians chose not to encourage the voluntary trial proposed by the government authority. This goes against the principle of effective implementation of change: all stakeholders involved (in particular farmers and veterinarians in the EP) must feel implicated, support the change and give it a sustainable meaning and purpose [8, 9]. The position of veterinarians and their ongoing relationships with farmers reveal their implication in the management of the farm (its trajectory and its future), their financial dependence, and their fear of client loss, which have been reported in previous studies [39,40]. In the context of bTB, a major challenge demands stakeholder motivation and engagement: Dorn and Mertig (2005) outlined a significant relationship between support for the goal of bTB eradication and the belief that such a goal is possible [64]. Applying this to the EP, we assume veterinarians lost confidence in the objectives of the trial due to its consequences on cattle, although they all expressed the need for a change in screening procedures.

Our analysis demonstrated that, most of the time, the implementation of the EP did not negatively affect the relationship between farmers and veterinarians. However, we cannot ignore that sometimes (for 3 farmers out of 11), the EP constituted a source of tension between them and could damage their mutual relationship of trust [59, 60]. According to the professional trade magazines [36], in some areas of France tensions had already heightened between the groups on the grounds of the cost of prophylactic measures. Further weakening of this relationship would be a particular cause for concern because this partnership constitutes a major pillar of animal health for the state veterinary services. Alongside farmers, veterinarians play a prominent role in animal health and food safety policies, particularly in terms of surveillance and animal disease prevention and control [40, 65, 66]. More and Good (2006) outlined the benefit of awareness campaigns on the bTB infectious and control measures to be taken in Ireland [67]. In the EP context, and more broadly in bTB screening, such campaigns would be useful in France to advance herd owner understanding about bTB and the tests used, and to support communication by veterinarians at the field level.

#### Targeting the interests of stakeholders: A driving factor in encouraging participation

To motivate stakeholders, to generate or raise their interest, and to ensure their subsequent involvement, relevant and necessary criteria must be identified at an early stage to lead stakeholders to change their way of working [8]. In a cross-cutting manner, stakeholders can either upset the proposed change or make it happen [56]. In terms of the proposed change in disease control, the meaning stakeholders give to actions is crucial [8]. The process of change itself relies on the consent and commitment of stakeholders, who require certain legitimacy [68]. Consequently, in our case, considering the EP as a temporary change in the bTB screening protocol in cattle, the main issue is to identify the key components that could help avert any opposition to the proposed change.

Our study demonstrated that the optional maintenance of good health status of the herd (that allowed movements of cattle in France despite the presence of a suspected case) was not a sufficient incentive to encourage farmer participation. In addition, the diagnostic slaughters, which were more numerous than expected, constituted an obvious element of rejection. However, change (here in the management protocol) results from at least two principles: autonomy of stakeholders and the legitimacy they give to the actions and decisions relating to the change [8, 56]. The second point had not been met during the EP; farmers did not recognise its objectives as being fair and rational [8]. Farmers indicated that they may have renewed their participation in the EP the next year on the condition that the EP did not impose additional constraints (such as diagnostic slaughter) in the process of managing a suspected bTB case. However, this relief measure would have jeopardised the scientific objectives.

## Conclusion

According to the Bayesian paradigm applied to a sociological purpose, local prior knowledge should be considered in the design and communication of an experimental protocol to avoid major difficulties, participation refusal and common pitfalls. Our qualitative study sheds light on the factors leading farmers to participate in or to refuse to join an animal health trial. Farmers initially had a good opinion of the experimental protocol and the IFN test, hoping for relief in the management of suspected bTB cases. This incited them to join the trial but the consequences of their participation, and their lack of knowledge about the trial and its goals, contributed to their rejection of the trial. Relationships between veterinarians and farmers played a major role in the decision-making process. Our findings highlighted that trials cannot succeed in achieving change without appealing to participants’ consent and appropriation, and this acceptance is created through stakeholder interactions, participation and education. Participatory methods seem relevant to address this challenge. Several recommendations, including revising the documents used to present the EP, transforming scientific outputs or text into clear visuals that are quickly understood by farmers, and providing adequate communication tools, support and accurate information to the other field stakeholders involved, may improve participation in an animal health trial. Even though the divergence between practice and the scientific world might be substantial in infectious disease research, we strongly believe that appropriate incentives and measures can minimise the practice-theory gap and facilitate interdisciplinary communication.

## Supporting information

S1 Fig Diagrams illustrating the current bTB screening protocol, the experimental protocol and the protocol expected to replace the current one. (DOCX)

S1 Table Table illustrating topics and underlying topics from interview guides with farmers, veterinarians, the departmental testing laboratory agent, GTV representatives, GDS representatives and veterinary services agents. (DOCX)

S2 Table Table providing translations of the adjectives given in Table 5. (DOCX)

S3 Table Table providing translations of the adjectives given in Table 6. (DOCX)

S4 Table Indicative table for translations presented in the paper: French transcribed quotes with their English translation. (DOCX)

## References

[1] Domingo M, Vidal E, Marco A. Pathology of bovine tuberculosis. Res Vet Sci. 2014; 97, Supplement: S20–9. 24731532 10.1016/j.rvsc.2014.03.017

[2] LoBue PA, Enarson DA, Thoen CO. Tuberculosis in humans and animals: an overview. Int J Tuberc Lung Dis Off J Int Union Tuberc Lung Dis. 2010; 14(9): 1075–8. 20819249

[3] de la Rua-Domenech R, Goodchild AT, Vordermeier HM, Hewinson RG, Christiansen KH, Clifton-Hadley RS. Ante mortem diagnosis of tuberculosis in cattle: a review of the tuberculin tests, gamma-interferon assay and other ancillary diagnostic techniques. Res Vet Sci. 2006; 81(2): 190–210. doi: 10.1016/j.rvsc.2005.11.005 16513150 10.1016/j.rvsc.2005.11.005

[4] Ryan TJ, Buddle BM, De Lisle GW. An evaluation of the gamma interferon test for detecting bovine tuberculosis in cattle 8 to 28 days after tuberculin skin testing. Res Vet Sci. 2000; 69(1): 57–61. doi: 10.1053/rvsc.2000.0386 10924395 10.1053/rvsc.2000.0386

[5] Praud A, Boschiroli ML, Meyer L, Garin-Bastuji B, Dufour B. Assessment of the sensitivity of the gamma-interferon test and the single intradermal comparative cervical test for the diagnosis of bovine tuberculosis under field conditions. Epidemiol Infect. 2014; 1–10. 10.1017/S0950268814000338 PMC9206784 24576504

[6] Anonymous. Note de service DGAL/SDSPA/N2014-864 du 28 octobre 2014. Modification de la note de service DGAL/SDSPA/N2013-8162 relative au protocole expérimental d’évaluation de l’interféron gamma [Internet]. 2014 [Updated 2014 Oct 10; cited 2016 Aug 01]. https://info.agriculture.gouv.fr/gedei/site/bo-agri/instruction-2014-864

[7] Praud A, Boireau C, Dufour B. Short communication: Sensitivity of γ-interferon test used in series after tuberculin test to detect bovine tuberculosis. Vet Rec. 2016; 179(7): 174. 10.1136/vr.103803 27402595

[8] Bernoux P. Sociologie du changement dans les entreprises et les organisations. Points; 2010 368 p.

[9] Crozier M, Friedberg E. L’acteur et le système: Les contraintes de l’action collective. Points; 2014 500 p.

[10] Anonymous. Note de service DGAL/SDSPA/N2013-8162 du 8 octobre 2013: Protocole expérimental d’évaluation de l’Interféron Gamma [Internet]. 2013 [Updated 2013 Oct 08; cited 2016 Aug 01]. http://agriculture.gouv.fr/sites/minagri/files/documents/pdf/Protocole_evaluation_interferon_DGALN20138162_cle018ba9.pdf

[11] Collingridge DS, Gantt EE. The quality of qualitative research. Am J Med Qual Off J Am Coll Med Qual. 2008; 23(5): 389–95. 10.1177/1062860608320646 18820144

[12] Ferréol G. Dictionnaire de sociologie. 4th edition Armand Colin; 2011 332 p.

[13] Onwuegbuzie AJ, Leech NL. A call for qualitative power analyses. Qual Quant. 2007; 41(1): 105–21.

[14] Beaud S, Weber F. Guide de l’enquête de terrain: Produire et analyser des données ethnographiques. New. Ed. La Découverte; 2003 356 p.

[15] Given L. Qualitative research in evidence-based practice: a valuable partnership. Libr Hi Tech. 2006; 24(3): 376–86.

[16] Blanchet A, Gotman A. L’entretien: L’enquête et ses méthodes. 2nd edition Armand Colin; 2010 128 p.

[17] Kaufmann J-C. L’entretien compréhensif—L’enquête et ses méthodes. 3rd edition Armand Colin; 2011 128 p.

[18] Britten N. Qualitative interviews in medical research. BMJ. 1995; 311(6999): 251–3. 7627048 10.1136/bmj.311.6999.251 PMC2550292

[19] Ghiglione R, Matalon B. Les enquêtes sociologiques: Théories et pratique. 6th edition Armand Colin; 1998 304 p.

[20] Becker HS. Les ficelles du métier Comment conduire sa recherche en sciences sociales. La Découverte; 2002 360 p.

[21] DiCicco-Bloom B, Crabtree BF. The qualitative research interview. Med Educ. 2006; 40(4): 314–21. doi: 10.1111/j.1365-2929.2006.02418.x 16573666 10.1111/j.1365-2929.2006.02418.x

[22] Braun V, Clarke V. Using thematic analysis in psychology. Qual Res Psychol. 2006; 3(2): 77–101.

[23] Graneheim UH, Lundman B. Qualitative content analysis in nursing research: concepts, procedures and measures to achieve trustworthiness. Nurse Educ Today. 2004; 24(2): 105–12. doi: 10.1016/j.nedt.2003.10.001 14769454 10.1016/j.nedt.2003.10.001

[24] Braun V, Clarke V. What can « thematic analysis » offer health and wellbeing researchers? Int J Qual Stud Health Well-Being. 2014; 9:26152 doi: 10.3402/qhw.v9.26152 25326092 10.3402/qhw.v9.26152 PMC4201665

[25] Bardin L. L’analyse de contenu. 2nd edition Presses Universitaires de France; 2013 320 p.

[26] Mucchielli R. L’analyse de contenu: des documents et des communications. 9th edition ESF Editeur; 2006 223 p.

[27] Mukamurera J, Lacourse F, Couturier Y. Des avancées en analyse qualitative: pour une transparence et une systématisation des pratiques. Rech Qual. 2006; 26(1): 110–38.

[28] Mays N, Pope C. Rigour and qualitative research. BMJ. 1995; 311(6997):109–12. 7613363 10.1136/bmj.311.6997.109 PMC2550154

[29] Olivier De Sardan J-PO. La rigueur du qualitatif: Les contraintes empiriques de l’interprétation socio-anthropologique. Editions Academia; 2008 365 p.

[30] Tracy SJ. Qualitative quality: Eight a"big-tent" criteria for excellent qualitative research. Qual Inq. 2010; 16(10): 837–51.

[31] Guest G, Bunce A, Johnson L. How Many Interviews Are Enough? An Experiment with Data Saturation and Variability. Field Methods. 2006; 18(1): 59–82.

[32] Malterud K. Qualitative research: standards, challenges, and guidelines. Lancet Lond Engl. 2001; 358(9280): 483–8. 10.1016/S0140-6736(01)05627-6 11513933

[33] Al-Busaidi ZQ. Qualitative Research and its Uses in Health Care. Sultan Qaboos Univ Med J. 2008; 8(1): 11–9. 21654952 PMC3087733

[34] Christley RM, Perkins E. Researching hard to reach areas of knowledge: qualitative research in veterinary science. Equine Vet J. 2010; 42(4): 285–6. doi: 10.1111/j.2042-3306.2010.00074.x 20525043 10.1111/j.2042-3306.2010.00074.x

[35] Van Campenhoudt L, Quivy R. Manuel de recherche en sciences sociales. 4th edition Dunod; 2011 272 p.

[36] Boireau C. Etude des caractéristiques intrinsèques du test interféron gamma utilisé en série suite à une intradermotuberculination dans le cadre du dépistage de la tuberculose bovine en France et enquête sociologique auprès des acteurs locaux [Thèse de Doctorat Vétérinaire]. [Maisons-Alfort]: Faculté de médecine de Créteil; 2015.

[37] Autissier D, Moutot J-M. Méthode de conduite du changement. 3rd edition Dunod; 2013 256 p.

[38] Guta S, Casal J, Napp S, Saez JL, Garcia-Saenz A, Perez de Val B, et al Epidemiological investigation of bovine tuberculosis herd breakdowns in Spain 2009/2011. PloS One. 2014; 9(8):e104383 doi: 10.1371/journal.pone.0104383 25127254 10.1371/journal.pone.0104383 PMC4134210

[39] Enticott G. The local universality of veterinary expertise and the geography of animal disease. Trans Inst Br Geogr. 2012; 37(1): 75–88.

[40] Meskell P, Devitt C, More SJ. Challenges to quality testing for bovine tuberculosis in Ireland; perspectives from major stakeholders. Vet Rec. 2013; 173(4): 94 doi: 10.1136/vr.101676 23893590 10.1136/vr.101676

[41] Henning J, Hla T, Meers J. Interdisciplinary communication of infectious disease research–translating complex epidemiological findings into understandable messages for village chicken farmers in Myanmar. SpringerPlus. 2014; 3:726 doi: 10.1186/2193-1801-3-726 25674462 10.1186/2193-1801-3-726 PMC4320238

[42] Decaudin J-M, Igalens J, Waller S. La communication interne, stratégies et techniques. 3rd edition Dunod; 2013 224 p.

[43] Bernoux P. Le changement dans les organisations. Entre structures et interactions. Relat Ind Ind Relat. 2002; (57–1): 77–99.

[44] Dent EB, Goldberg SG. Challenging “Resistance to Change”. J Appl Behav Sci. 1999; 35(1): 25–41.

[45] Lawrence PR. How to deal with resistance to change. Harv Bus Rev. 1969; 47(1): 4–6.

[46] Alders RG, Bagnol B. Effective communication: the key to efficient HPAI prevention and control. Worlds Poult Sci J. 2007; 63(01): 139–147.

[47] Jones B, Araba A, Koskei P, Letereuwa S. Doing it for themselves: how communities developed messages and communication methods for rinderpest eradication in southern Sudan. Particip Learn Action Ser. 2002; 45:5.

[48] Coch L, French JRP. Overcoming resistance to change Hum Relat. 1948; 512–32.

[49] Pretty JN. Participatory learning for sustainable agriculture. World Dev. 1995; 23(8): 1247–63.

[50] Catley A, Alders RG, Wood JLN. Participatory epidemiology: approaches, methods, experiences. Vet J Lond Engl 1997. 2012; 191(2): 151–60. 10.1016/j.tvjl.2011.03.010 21856195

[51] van Dijk L, Hayton A, Main DCJ, Booth A, King A, Barrett DC, et al Participatory Policy Making by Dairy Producers to Reduce Anti-Microbial use on Farms. Zoonoses Public Health. 2016; doi: 10.1111/zph.12329 28026910 10.1111/zph.12329

[52] Catley A, Leyland T. Community participation and the delivery of veterinary services in Africa. Prev Vet Med. 2001; 49(1–2): 95–113. 11267692 10.1016/s0167-5877(01)00171-4

[53] Sungirai M, Moyo DZ, De Clercq P, Madder M. Communal farmers’ perceptions of tick-borne diseases affecting cattle and investigation of tick control methods practiced in Zimbabwe. Ticks Tick-Borne Dis. 2016; 7(1): 1–9. doi: 10.1016/j.ttbdis.2015.07.015 26234572 10.1016/j.ttbdis.2015.07.015

[54] Nantima N, Davies J, Dione M, Ocaido M, Okoth E, Mugisha A, et al Enhancing knowledge and awareness of biosecurity practices for control of African swine fever among smallholder pig farmers in four districts along the Kenya-Uganda border. Trop Anim Health Prod. 2016; 48(4): 727–34. doi: 10.1007/s11250-016-1015-8 26922740 10.1007/s11250-016-1015-8

[55] Azhar M, Lubis AS, Siregar ES, Alders RG, Brum E, McGrane J, et al Participatory disease surveillance and response in Indonesia: strengthening veterinary services and empowering communities to prevent and control highly pathogenic avian influenza. Avian Dis. 2010; 54(1 Suppl): 749–53. doi: 10.1637/8713-031809-Reg.1 20521726 10.1637/8713-031809-Reg.1

[56] Kotter JP. Leading change: why transformation efforts fail. Harv Bus Rev. 1995;59–67.

[57] Mathevet P. Perception et attentes de l’éleveur bovin concernant le rôle du vétérinaire. In: GTV. Recueil des Journées Nationales des GTV, De l'urgence au conseil: le vétérinaire, partenaire de choix de l'éleveur de demain. Nantes; 2005. p. 73–81.

[58] Lam TJGM, Jansen J, van den Borne BHP, Renes RJ, Hogeveen H. What veterinarians need to know about communication to optimise their role as advisors on udder health in dairy herds. N Z Vet J. 2011; 59(1): 8–15. doi: 10.1080/00480169.2011.547163 21328152 10.1080/00480169.2011.547163

[59] Alarcon P, Wieland B, Mateus ALP, Dewberry C. Pig farmers’ perceptions, attitudes, influences and management of information in the decision-making process for disease control. Prev Vet Med. 2014; 116(3): 223–42. doi: 10.1016/j.prevetmed.2013.08.004 24016600 10.1016/j.prevetmed.2013.08.004

[60] Dean WR, Mcintosh WA, Morgan Scott H, Barling KS. The role of trust and moral obligation in beef cattle feed-lot veterinarians’ contingent adoption of antibiotic metaphylaxis recommendations. Int Jrnl Soc Agr Food. 2011; 18(2): 104–20.

[61] Richens IF, Hobson-West P, Brennan ML, Lowton R, Kaler J, Wapenaar W. Farmers’ perception of the role of veterinary surgeons in vaccination strategies on British dairy farms. Vet Rec. 2015; 177(18): 465 doi: 10.1136/vr.103415 26530434 10.1136/vr.103415 PMC4697308

[62] Garforth C. Livestock keepers’ reasons for doing and not doing things which governments, vets and scientists would like them to do. Zoonoses Public Health. 2015; 62 (1 Suppl): 29–38. 10.1111/zph.12189 25903493

[63] Humblet M-F, Walravens K, Salandre O, Boschiroli ML, Gilbert M, Berkvens D, et al Monitoring of the intra-dermal tuberculosis skin test performed by Belgian field practitioners. Res Vet Sci. 2011; 91(2): 199–207. doi: 10.1016/j.rvsc.2010.12.004 21208632 10.1016/j.rvsc.2010.12.004

[64] Dorn ML, Mertig AG. Bovine tuberculosis in Michigan: stakeholder attitudes and implications for eradication efforts. Wildl Soc Bull. 2005; 33(2): 539–52.

[65] O’Rourke D, Blake N. Veterinary involvement in TB control. Vet Rec. 2014; 174(12): 307. 10.1136/vr.g2231 24652848

[66] Palmer MV, Waters WR. Bovine tuberculosis and the establishment of an eradication program in the United States: role of veterinarians. Vet Med Int. 2011; 2011:816345 doi: 10.4061/2011/816345 21647341 10.4061/2011/816345 PMC3103864

[67] More SJ, Good M. The tuberculosis eradication programme in Ireland: a review of scientific and policy advances since 1988. Vet Microbiol. 2006; 112(2–4): 239–51. doi: 10.1016/j.vetmic.2005.11.022 16337345 10.1016/j.vetmic.2005.11.022

[68] Paillé P. Changement organisationnel et mobilisation des ressources humaines. L’Harmattan; 2003 258 p.

