# Supplementary material for: The importance of communication in promoting voluntary participation in an experimental trial: A qualitative study based on the assessment of the gamma-interferon test for the diagnosis of bovine tuberculosis in France
Source: PLoS One. 2017 Oct 3;12(10):e0185799. doi: 10.1371/journal.pone.0185799 (PMC5626495; doi:10.1371/journal.pone.0185799)

**S1 Figure. Diagrams illustrating the current bTB screening protocol, the experimental protocol and the protocol expected to replace the current one**


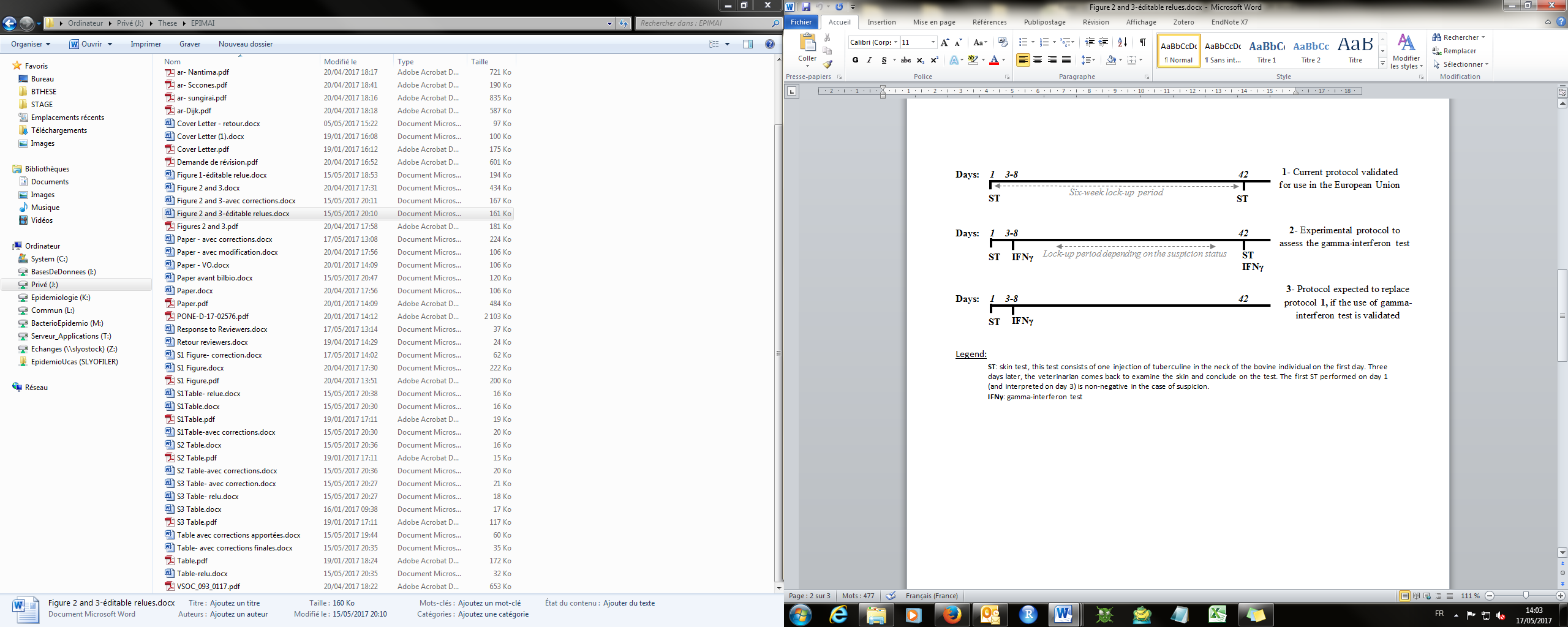

Supplement: S1 Fig — (DOCX) [file pone.0185799.s001.docx]
