# Supplementary material for: The importance of communication in promoting voluntary participation in an experimental trial: A qualitative study based on the assessment of the gamma-interferon test for the diagnosis of bovine tuberculosis in France
Source: PLoS One. 2017 Oct 3;12(10):e0185799. doi: 10.1371/journal.pone.0185799 (PMC5626495; doi:10.1371/journal.pone.0185799)
Supplement: S1 Table — (DOCX) [file pone.0185799.s002.docx]

**S1 Table. Table illustrating topics and underlying topics from interview guides with farmers, veterinarians, the departmental testing laboratory agent, GTV representatives, GDS representatives and veterinary services agents.**

| **Topics** | **Stakeholder** | **Underlying topics** |
| --- | --- | --- |
| **Introduction** | **All stakeholders** | - Presentation of the interviewer  - Presentation of the aim and background of the study  - Modalities of the study (methodology, no right or wrong answer, personal views and experiences are valued, the need to audio-record, ensure confidentiality)  - Verbal consent  - Date and location of the interview |
| **Participant background** | **All stakeholders** | - Name  - Gender  - Age - years of practice  - Years spent in Ardennes  - Years of participation in the experimental protocol (EP) |
|  | **Farmer** | - Herd size  - Cattle breed  - Brief description of the farm (size, workers)  - Other activities/occupation |
|  | **Veterinarian** | - Activity  - Brief description of the clientele |
|  | **Laboratory agent** | - Brief description of the laboratory  - Role in laboratory activities |
|  | **GTV representative** | - Brief description of representatives’ activities  - Other activities/occupation |
|  | **GDS representative** |  |
|  | **Veterinary services agent** | - Role in veterinary services activities |
| **Opening question** | **All stakeholders** | - What led you to join the experimental protocol? |
| **Knowledge regarding the EP** | **All stakeholders** | - Ability to describe the experimental protocol  - Knowledge regarding the purpose of the experiment |
| **Managing temporary change** | **All stakeholders** | - Presentation of the experimental protocol to stakeholders (who, when, where)  - Information received: relevance, quantity, quality  - Information relayed in the field (who, when, how)  - Active research of further information (what, why, where, when, difficulties met)  - Opinion and view on the organisation and the management of the experimental protocol  - Relationships with other stakeholders (creation, modification, evolution) |
|  | **Veterinarian** | - Training (when, where, by whom)  - Opinion and view about the training  - Change in practice activities induced by the EP |
|  | **Laboratory agent** | - Technical training (when, where, by whom)  - Opinion and view about the technical training  - Change in laboratory activities induced by the EP |
|  | **GTV representative** | - Training received (when, where, by whom)  - Training and information provided (when, where, to whom, how)  - Opinion and view about the training received and provided  - Change in activities induced by the EP  - Management changes between the two campaigns |
|  | **GDS representative** |  |
|  | **Veterinary services agent** |  |
| **Motivation and interest behind participation** | **All stakeholders** | - Factors that influenced participation or support for the EP  - Personal interest  - Third-party opinions or arguments that influenced the decision  - Changes in viewpoint |
|  | **Veterinarian** | - Advice provided to farmers |
|  | **Laboratory agent** | - Development of laboratory activities |
|  | **GTV representative** | - Advice provided to veterinarian |
|  | **GDS representative** | - Advice provided to farmers |
|  | **Veterinary services agent** | - Advice provided to farmers and veterinarians |
| **Drawbacks and obstacles to participation** | **All stakeholders** | - Factors that influenced refusal to participate or to support the EP  - Reasons for dissatisfaction  - Third-party opinions or arguments that influenced the decision  - Changes in viewpoint |
|  | **Veterinarian** | - Practical and technical difficulties met |
|  | **Laboratory agent** |  |
|  | **Veterinary services agent** | - Practical difficulties met  - Way to counter the drop off in participation |
| **Knowledge of the gamma-interferon test** | **All stakeholders** | - Ability to talk about the test and present it  - First impressions of it (drawbacks, advantages, comparisons with other tests)  - Changes in viewpoint  - Issues on its use for screening bovine tuberculosis |
| **Issues regarding changes to the current control** | **All stakeholders** | - Opinion about the management procedure for suspect bovine tuberculosis cases.  - Opinion and feelings on the lock-up period  - Issues at stake for shortening the lock-up period |
|  | **Veterinarian** | - Suspected impact of the modification of the current control procedure (on their activity, on the relationships between stakeholders)  - Ways to improve the current control procedure |
|  | **Laboratory agent** |  |
|  | **GTV representative** |  |
|  | **GDS representative** |  |
|  | **Veterinary services agent** |  |
| **Concluding remarks** | **All stakeholders** | - General opinion on the trial  - Please list five adjectives to describe the experimental protocol or the gamma-interferon test?  - Ways to improve the trial  - Others remarks  - Words of thanks |
