# Supplementary material for: The importance of communication in promoting voluntary participation in an experimental trial: A qualitative study based on the assessment of the gamma-interferon test for the diagnosis of bovine tuberculosis in France
Source: PLoS One. 2017 Oct 3;12(10):e0185799. doi: 10.1371/journal.pone.0185799 (PMC5626495; doi:10.1371/journal.pone.0185799)
Supplement: S2 Table — (DOCX) [file pone.0185799.s003.docx]

**S2 Table. Table providing translations of the adjectives given in Table 5**

| **Adjectives in French** | **Adjectives in English** |
| --- | --- |
| pas adapté | *unsuitable* |
| long | *long* |
| lourd | *cumbersome* |
| rigide | *rigid* |
| non-réaliste | *unrealistic* |
| mal engagé | *poorly initiated* |
| contraignant | *strict* |
| compliqué | *complicated* |
| pas clair | *unclear* |
| pas convainquant | *unconvincing* |
| inquiétant | *worrying* |
| bloquant | *interfering* |
| pénalisant | *punitive* |
| strict | *strict* |
| mal-vécu | *upsetting* |
| couteux | *costly* |
| onéreux | *expensive* |
| mal adapté | *unadapted* |
| chronophage | *time-consuming* |
| lent | slow |
| trop mobilisateur | *too heavy* |
| perfectible | *perfectible* |
| peu accessible | *not easy to understand* |
| exigeant | *demanding* |
| expérimental | *experimental* |
| très technique | *very technical* |
| non conclusif | *inconclusive* |
| obscure | *obscure* |
| confus | *confusing* |
| flou | *vague* |
| nul | *lousy* |
| améliorable | *improvable* |
| risqué | *risky* |
| peu acceptable | *not easily acceptable* |
| rejeté | *rejected* |
| déconseillé | *inadvisable* |
| stressant | *stressful* |
| unilatéral | *unilateral* |
| spoliateur | *confiscatory* |
