# Supplementary material for: The importance of communication in promoting voluntary participation in an experimental trial: A qualitative study based on the assessment of the gamma-interferon test for the diagnosis of bovine tuberculosis in France
Source: PLoS One. 2017 Oct 3;12(10):e0185799. doi: 10.1371/journal.pone.0185799 (PMC5626495; doi:10.1371/journal.pone.0185799)
Supplement: S3 Table — (DOCX) [file pone.0185799.s004.docx]

**S3 Table. Table providing translations of the adjectives given in Table 6**

| **Adjectives in French** | **Adjectives in English** |
| --- | --- |
| sécuritaire | *safe* |
| rapide | *fast* |
| simple | *simple* |
| précis | *accurate* |
| nouveau | *new* |
| pratique | *convenient* |
| supportable | *bearable* |
| facile | *easy* |
| non-impliquant | *objective* |
| intéressant | *interesting* |
| bénéfique | *beneficial* |
| cher | *expensive* |
| couteux | *costly* |
| pas pratique | *inconvenient* |
| inefficace | *ineffective* |
| contraignant | *restrictive* |
| compliqué | *complicated* |
| mauvais | *bad* |
| douteux | *doubtful* |
| approximatif | *approximate* |
| aléatoire | *arbitrary* |
| pas fiable | *unreliable* |
| peu fiable | *not really reliable* |
| imprévisible | *unpredictable* |
| inutile | *useless* |
| améliorable | *improvable* |
| inadapté | *unsuitable* |
| confus | *confusing* |
| mitigé | *mixed* |
| affinable | *adaptable* |
