# Supplementary material for: The importance of communication in promoting voluntary participation in an experimental trial: A qualitative study based on the assessment of the gamma-interferon test for the diagnosis of bovine tuberculosis in France
Source: PLoS One. 2017 Oct 3;12(10):e0185799. doi: 10.1371/journal.pone.0185799 (PMC5626495; doi:10.1371/journal.pone.0185799)
Supplement: S4 Table — (DOCX) [file pone.0185799.s005.docx]

**S4 Table. Indicative table for translations presented in the paper: French transcribed quotes with their English translation.**

| **French quotes** | **English quotes** |
| --- | --- |
| *« L’intradermo pour moi c’est super biaisé, enfin moi j’trouve, parce que voilà, c’est une question de millimètre au cutimètre. Après quand elles [les vaches] sont bien attachées ça va, mais quand elles passent au couloir et qu’elles sont pressées… Et puis en fonction du pli de peau qu’on fait : c’est biaisé, c’est fort biaisé je trouve !».* | *“For me, skin tests are really biased, because it is a matter of [just a few] millimetres with the caliper. Afterwards, when they [cows] are well tied-up, it’s OK, but when they go through the containment corridor* *and they are stressed… And it depends on the fold of skin we choose: it’s biased, it’s really biased I think”* |
| « *On ne peut pas tout comprendre ! Nous on est du côté des scientifiques, et on doit expliquer aux gens […] qu’on est en face d’une des rares maladies où c’est l’enfer du diagnostic ! Le problème, il est là : c’est l’enfer du diagnostic ! [Ton exaspéré]* » | *“We can’t understand everything! We [meaning veterinarians] are on the side of the scientists and we have to explain to people […] that we are dealing with one of the rare diseases where the diagnosis is a nightmare! That’s the problem: the diagnosis is a nightmare! [Exasperated tone]”* |
| *« On a eu longuement les éleveurs au téléphone, mais le problème c’est que le protocole c’est assez compliqué et pour l’expliquer au téléphone…[haussement de sourcil] »* | *“We spent long time on the phone with farmers, but the problem is that the protocol [the EP] is quite complicated, let alone explaining it on the phone …[Raised eyebrows]”* |
| *« C’est trop vague, il y a trop de texte* » | *“It’s too vague, there is too much text”* |
| *« Le formulaire d’engagement a beaucoup trop de texte »* | “*The enrolment form is too wordy*” |
| *« Dans le cadre de ce protocole vous pourrez bénéficier de mesures d’allègement vis-à-vis de la suspension de qualification et de mesures plus ciblées d’abattages diagnostiques* » | “*In this protocol, you can benefit from relief measures with regard to the demotion of the good health status qualification, and you can benefit from more targeted measures regarding the slaughter of suspect animals*” |
| *« C’est un résultat où on n’a pas trop envie de se mouiller ! [Rires jaunes] Non c’est ça, voilà il y a quelque chose qui réagit, il y a un autre qui ne réagit pas, on ne sait pas. Et même nous on a du mal à l’expliquer de manière simple aux éleveurs »* | *“It’s an outcome we don’t want to stick our necks out for! [Forced laughter] No, that’s it, one test reacts and another doesn’t, we don’t know. And even for us it’s difficult to explain it simply to farmers”* |
| *« C’est difficilement compris donc difficilement accepté »* | “*It’s hard to understand, it’s difficult to accept”* |
| *« Il faudrait mettre peut-être un niveau, par exemple […] un niveau vert, orange, rouge. Peut-être parce que non-conclusif, voilà ça veut dire qu’on n’a pas de conclusion. Et les gens et moi-même au tout début, je le prenais comme ininterprétable. Mais finalement là [sous-entendu avec un barème de couleur] comme on le voit ce serait un douteux : il y a quelque chose qui a bougé et quelque chose qui n’a pas bougé »* | “*Maybe we should use levels, for instance […] a green/orange/red level. Perhaps because an inconclusive outcome means there is no conclusion. And people, including me at the very beginning, thought it meant uninterpretable. But here [with a colour scale], we see it as a doubtful result: one thing has been modified, the other thing didn’t change”* |
| *« Pour nous, c’est l’intérêt des éleveurs avant tout »* | “*For us, the interest of farmers comes first*” |
| *« Je pouvais dire non [sous-entendu au PEE-IFNγ]… la décision finale c’est moi qui l’avais, j’ai choisi de suivre leur avis*» | *“I could have said no [meaning to the EP]… the final decision was mine. I chose to follow their advice*” |
| *« Finalement il y a une énorme frustration des éleveurs parce qu’ils pensaient que ça allait faciliter leur situation »* | *“In the end, farmers are extremely frustrated because they thought it would improve their situation*” |
